# Ferritin in glioblastoma

**DOI:** 10.1038/s41416-020-0808-8

**Published:** 2020-03-23

**Authors:** Heidi Jaksch-Bogensperger, Sabine Spiegl-Kreinecker, Paolo Arosio, Peter Eckl, Stefan Golaszewski, Yvonne Ebner, Rahman Al-Schameri, Peter Strasser, Serge Weis, Nikolaus Bresgen

**Affiliations:** 1 Department of Biosciences, University of Salzburg, Hellbrunnerstrasse 34, Salzburg, A-5020 Austria; 2 Department of Obstetrics and Gynaecology, Salzburger Landeskliniken (SALK), Paracelsus Medical University Salzburg (PMU), Clinical Research Center, Salzburg, A-5020 Austria; 3 University Hospital of Neurology, Christian-Doppler-Klinik, Salzburger Landeskliniken (SALK), Paracelsus Medical University Salzburg (PMU), Salzburg, A-5020 Austria; 4 Department of Neurosurgery, Kepler University Hospital GmbH, Johannes Kepler University, Linz, A-4020 Austria; 5 Laboratory of Molecular Biology, Department of Molecular and Translational Medicine, University of Brescia, Brescia, I-25123 Italy; 6 Department of Neurosurgery, Christian Doppler Klinik, Salzburger Landeskliniken (SALK), Paracelsus Medical University Salzburg (PMU), Salzburg, A-5020 Austria; 7 Institute for Medical and Chemical Laboratory Diagnostics, Salzburger Landeskliniken (SALK), Paracelsus Medical University Salzburg (PMU), Salzburg, A-5020 Austria; 8 Department of Neuropathology, Kepler University Hospital GmbH, Johannes Kepler University, Linz, A-4020 Austria

**Keywords:** CNS cancer, Diagnostic markers

## Abstract

Elevated levels of serum ferritin (SF) are observed in several types of cancer; however, little is known on the association between ferritin and glioma, the most frequent type of human primary brain tumour. Here we report that GBM patients show significantly increased pre-surgical SF levels (i.e. ferritinaemia) within the SF reference range and a marked ferritin immunoreactivity of resected tumour tissue. Our findings account for an indirect association between ferritin synthesis in glioma-tissue and altered SF levels, which limits the clinical value of SF as a tumour marker in glioma. Importantly, we show for the first time that GBM-derived glioma cells release ferritin in vitro, which exerts an apoptosis-stimulating activity. Albeit the pathophysiologic context of apoptosis induction by a tumour-derived ferritin remains to be defined, our findings account for a distinct growth-regulatory role of these ferritin species in tumour biology.

## Background

Ferritin, a 450-kDa multimeric iron-storage protein, built from 24 heavy (FTH) and light (FTL) chains, is essential to cellular iron homoeostasis by regulating the intracellularlabileiron pool via its ferroxidase activity conferred by the FTH chain.^1^ In the brain, FTH-rich isoferritins predominate in neurons and oligodendrocytes, FTL-rich isoferritins in microglial cells and astrocytes in the corpus striatum.^2^ Serum ferritin (SF) levels are normally low, but may rise in diseased states, including cancer, leading to malignancy-associated ferritinaemia.^1,3^ Tumour cell-based ferritin release (e.g. neuroblastoma) is considered to be causal to rising SF levels, but other sources (e.g. cells of the tumour-surrounding stroma) have also been discussed.^1^ Still, the clinical and pathophysiological significance of ferritin in cancer is poorly defined, which holds particularly true for glial tumours. Only three studies have assessed ferritinaemia in gliomas by routine laboratory diagnostics comprising a total number of less than 60 patients,^4–6^ the biological background of glioma-associated ferritinaemia remaining elusive. Here, we provide evidence for an upregulated ferritin synthesis in glioma tissue, which is not directly associated with elevated SF levels. In addition, we show for the first time that tumour-derived ferritins are capable of stimulating apoptosis.

## Methods

The study was approved by the local research ethics committee Salzburg, Austria (415-EP/33/3-2008). Written informed consent was obtained from all patients. The study was performed in accordance with the Declaration of Helsinki. Pre-surgical serum samples and tumour tissue specimens from 18 GBM patients and 16 meningioma (WHO I) patients were investigated. Serum ferritin (SF) was quantified by employing the Tina-Quant-Ferritin assay (ROCHE, Germany) on a Hitachi 917 automatic analyser. Immunohistochemical analysis of formalin-fixed paraffin-embedded specimens of resected tumour tissue followed standard procedures by employing anti-human FTL-specific (LSBio LS-B4383) and FTH-specific (LSBio LS-C105404) antibodies. Determination of the ferritin-labelling index is outlined in Supplementary Fig. S1. The apoptosis-inducing activity of ferritin released from a primary human glioma cell line,^7^ as well as newborn mouse astrocytes, was investigated under serum-free conditions using a primary rat hepatocyte assay defined for assessing ferritin cytotoxicity.^8,9^ Statistical significance was examined by applying non-parametric tests (SF quantification using published gender-specific population medians^3^ as reference, immunohistochemical analysis) and Student’s double-sided *t*-test for independent samples (apoptosis induction) using SPSS version 24.

## Results

**Fig. 1:**
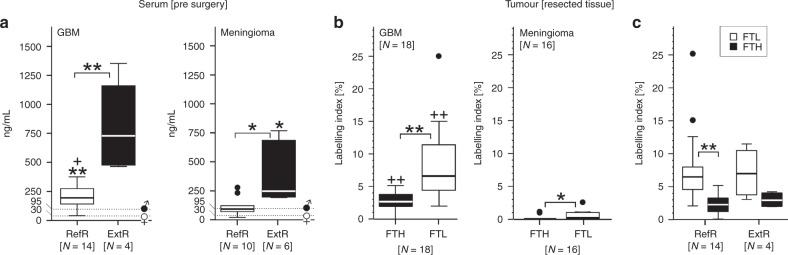
Ferritin in serum and resected tumour tissue of GBM and meningioma patients. **a** Based on SF quantification, patients were assigned to two cohorts: the RefR cohort (open boxes) showing SF levels inside the reference range (i.e. below the 95th percentile) of the TINA-Quant ferritin assay and the ExtR cohort (black boxes) with SF levels elevated above the 95th percentile. Dotted lines refer to the median reference levels reported for healthy male and female donors.^3^ Note that only a minority of GBM patients (ExtR cohort) showed very high SF levels (462–1355 ng/ml; median = 727 ng/mL), which is in line with existing data, with only 4 of a total of 57 glioma patients investigated so far, showing extremely high SF levels (Supplemental Table 1). Meningioma patients showed lower SF levels, which is significant (*p* > 0.05) for the RefR cohorts, and the highest SF value observed in meningioma patients (766 ng/mL) locates close to the median SF level of GBM–ExtR patients. **b** Immunohistochemistry of resected GBM and meningioma tissue evaluated by the labelling index (i.e. the percentage of immune-positive cells) demonstrates a significantly (*p* < 0.005) higher immunoreactivity for ferritin in GBM samples. In both types of tumour specimens, labelling for the FTL subunit was significantly (*p* < 0.05; *p* < 0.005) higher compared with FTH. **c** In GBM tissue, no differences of ferritin labelling were found between samples obtained from the RefR and ExtR cohort, the highest FTL labelling being observed in RefR cohort samples: 328 ng/mL (upper dot) and 206 ng/mL (lower dot). *p < 0.05; **p < 0.005 compared with the healthy reference population in (**a**) (Wilcoxon sign test for median difference based on gender-specific SF values) or as indicated (Mann–Whitney U exact test for independent samples for the comparison between the two patient cohorts in (**a**, **c**), and Wilcoxon signed-rank test for paired samples in **b**); ^+^*p* < 0.05; ^++^*p* < 0.005 compared with meningioma patients (Mann–Whitney U exact test for independent samples). The dots in meningioma RefR cohort (**a**) and (**b**) highlight statistical outliers. *N* refers to the number of investigated patients (**a**) or tumour specimens (**b**).

SF levels were significantly (*p* < 0.005) elevated in GBM and meningioma patients, in 22.2% (GBM) and 37.5% (meningioma) of the patients (ExtR cohort) exceeding the 95th percentile of the SF reference range (Fig. 1a; Supplementary Table 1). In GBM patients with SF levels within the reference range (RefR cohort), the ferritin concentration in serum was still significantly (*p* < 0.005) higher compared with a healthy reference population,^3^ as well as with the RefR cohort of meningioma patients (Fig. 1a). In good correspondence with this, resected tumour tissue from both patients immunoreacted with anti-ferritin antibodies, especially for the FTL subunit, GBM specimens showing significantly (*p* < 0.005) higher labelling indices (Fig. 1b; Supplementary Fig. 1). Interestingly, FTL and FTH-labelling indices were comparable between GBM specimens of the RefR and ExtR cohorts (Fig. 1c), which contrasts the significant variation of the corresponding SF levels (Fig. 1a). In line with this, SF levels and tissue FTL labelling of the GBM–RefR cohort showed a moderate, not significant correlation (Supplementary Fig. 2).

**Fig. 2:**
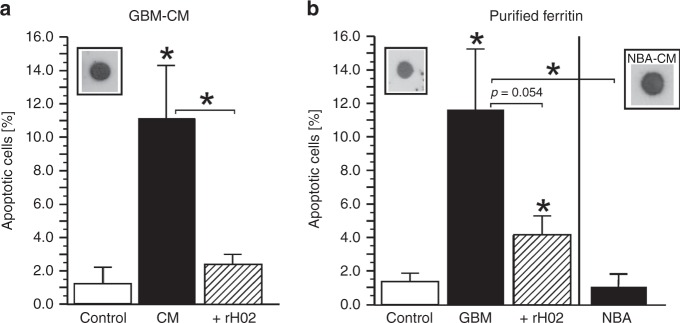
Cultured human glioma cells release an apoptosis-stimulating ferritin in vitro. **a** Ferritin is detectable in culture supernatants (GBM-CM) collected from human glioma-derived cells^7^ after 24 h of serum-free culture (inset: dot blot of GBM-CM [1.5 µg of total protein] immunoreacted with monoclonal anti-ferritin Ab rH02^15^). Upon 24 h of incubating primary rat hepatocytes under serum-free conditions in native GBM-CM [8.5 µg of total protein/mL], a significant (*p* < 0.05) shift of the percentage of apoptotic cells was observed (black bars), which was suppressed by anti-FTH antibody rH02 (hatched bar). **b** The ferritin was purified from GBM-CM according to a protocol described elsewhere^8^ (left inset: dot blot of purified ferritin [0.5 µg] immunoreacted with Ab rH02). Treatment of serum-free primary rat hepatocyte cultures for 48 h with 100 ng/ml of the purified GBM ferritin (GBM, closed bar) also had a significant (*p* < 0.05) apoptosis-stimulating effect that was suppressed by anti-FTH Ab rH02 (hatched bar). In contrast, a 48-h exposure of primary rat hepatocytes to 100 ng/mL of a ferritin purified from newborn mouse astrocyte culture supernatants^16^ had no effect (NBA). (**b** right inset: NBA culture supernatants [10 µg of total protein] immunoreacted with Ab rH02). Bars represent the mean ± SD of *N* ≥ 3 independent experiments. *P < 0.05 compared with the control or as indicated; Student’s double-sided *t-*test for independent samples.

Importantly, ferritin was also detectable in culture supernatants (GBM-CM) collected from a human glioblastoma-derived cell line (Fig. 2a). The GBM-CM as well as the ferritin purified thereof exerted a significant (*p* < 0.05) apoptosis-stimulating activity that was suppressed by neutralising anti-ferritin antibody Ab rH02 (Fig. 2a, b). In contrast, a ferritin species isolated from newborn mouse astrocyte cultures failed to stimulate apoptosis (Fig. 2b). This demonstrates for the first time that glial cells are able to release a ferritin isoform in vitro, which exerts a distinct apoptosis-inducing activity.

## Discussion

Our findings strongly support the assumption that glial tumours synthesise and secrete ferritin,^4,6^ which is causal to GBM-associated ferritinaemia. Moreover, the enhanced immunoreactivity of resected GBM tissue for the FTL subunit accounts for the synthesis of a FTL-type isoform, which corresponds well with the reported expression of FTL-type isoferritins in cultured glioblastoma-derived cells,^10^ and with similar findings in glioblastoma stem-like cells.^11^ Strikingly, we demonstrate for the first time an apoptosis-stimulating activity of GBM-derived isoferritins. Since this activity is not seen for the isoferritin released from cultured newborn astrocytes, it is conceivable that astrocyte transformation towards a malignant phenotype comes along with the synthesis and secretion of a different ferritin isoform that exerts a pro-apoptotic activity. Albeit the pathophysiological significance of this novel finding is elusive, earlier research has demonstrated that iron-mediated oxidative stress and lipid peroxidation plays a pivotal role in ferritin-mediated apoptosis.^12^ On the other hand, high cytosolic ferritin levels may confer cytoprotection by enhanced iron sequestration, and affecting stress-associated targets such as the GADD45A/JNK pathway.^10^ Notably, elevated iron requirements have been demonstrated for glioblastoma stem-like cells where an increased ferritin expression supposedly confers stable intracellular iron buffering.^11^

Interestingly, no significant correlation between tumour ferritin immunoreactivity and SF levels was found in the investigated GBM patients. Hence, the tumour-based ferritin release may not be directly connected with the circulating SF pool in GBM, which limits the use of serum ferritin as a useful tumour marker for gliomas. With respect to this, it is noteworthy that the blood–brain barrier (BBB) can persist in GBM tissue, which hinders the accessibility of chemotherapeutics to the tumour cells,^13^ and vice versa, it may also antagonise the exit of high-molecular-weight ferritin to the circulation. However, evidence exists that glioma cells themselves release soluble factors, such as vascular endothelial growth factor and hepatocyte growth factor, which promote BBB degradation.^14^ It is tempting to speculate on a similar effect conferred by the pro-apoptotic properties of glioma cell-derived isoferritins contributing to ferritinaemia in advanced GBM. The markedly high SF levels seen in the ExtR cohort of GBM patients could hypothetically reflect such a possibility. Similar contexts, although of less-severe outcome, may also apply to benign brain tumours, such as meningioma, which, as shown here, can also be accompanied by increased serum ferritin levels.

In conclusion, our findings account for a dual role of altered ferritin expression in glioma development: protecting the tumour cells by solid intracellular iron buffering, and upon release, acting as effector molecule in the tumour microenvironment.

## Supplementary information

Supplemental File

## Data Availability

Data are available from the corresponding author.
