# Supplementary material for: Ferritin in glioblastoma
Source: Br J Cancer. 2020 Mar 23;122(10):1441–4. doi: 10.1038/s41416-020-0808-8 (PMC7217840; doi:10.1038/s41416-020-0808-8)
Supplement: Supplementary file 1 — Supplemental File [file 41416_2020_808_MOESM1_ESM.pdf]

Supplemental Table 1 . Serum ferritin assessment – case summaries, reference ranges

Patient Cohort [1]		Serum ferritin [ng/mL] [2]	Assay [3]
GBM 63,5 (36 – 74 years)	All [18]	200,5 (35 – 1355)	Tina Quant®
	Men [16]	238,0 (96 – 1355)	
	Women [2]	52,0 (35 – 69)	
	RefR [14] [4]	190,0 (35 – 373)	
	ExtR [4] [4]	727,0 (462 – 1355)	
Meningioma 61,5 (45 – 73 years)	All [16]	154,5 (18 – 766)	Tina Quant®
	Men [5]	230,0 (106 – 682)	
	Women [11]	98,0 (18 – 766)	
**Reference Studies**			
Liu et al., 1991[8]	Astrocytoma WHO III [4]	1410,0 [5]	RIA
	GBM [3]	3816,6 [5]	RIA
Sato et al., 1998[9]	GBM [16]	20 – 200 [6]	RIA
Schwartzbaum & Cornwell, 2001[10]	GBM [34]	119,9 (69,0 – 281,9) [2]	RIA
	Control [35]	46,6 (29,6 – 103,0) [2]	
Lotz et al., 1999[11]	Healthy Men [120]	95,0 (29 – 321) [7]	N Latex Ferritin®
	Healthy Women [104]	30,0 (9 – 101) [7]	
	Healthy Men [120]	27 – 365 [7]	Tina Quant®
	Healthy Women [104]	13 – 148 [7]	
Roche-Diagnostics, 2012	Healthy Men [120]	30 – 400 [7]	Tina Quant®
	Healthy Women [104]	15 – 150 [7]	

[1] Diagnostic criteria followed the WHO classification of tumours of the central nervous system [Louis et al., 2016. WHO classification of tumours ofthe centralnervous system.4th Edition]. Indicated is the median age, age range and the number of investigated patients (in brackets).[2] If not otherwise stated SF values represent the median and range (minimum – maximum). [3] SF quantification method: RIA, radioimmunoassay (Microparticle Enzyme Immunoassay; Abbott); nepehelometric assay: N Latex Ferritin®, Cobas Tina Quant FERR4 ® (ROCHE Diagnostics). [4] Cohort defined by the SF values < (RefR) or > (ExtR) the 95th percentile of the Tina Quant FERR4 assay. [5] Value represents the mean; no measure of variance is given in the report. [6] Calculated range based on the reported CSF-ferritin levels and the CSF-ferritin/SF ratio. [7] Values are the median and 5th – 95th percentile of SF measurements.

[8] Liu YF, Li Q, Yang P, Wang WL, Liu JA. Ferritin in astrocytomas. Chin Med J (Engl). 1991;104(4):326-9. [9] Sato Y, Honda Y, Asoh T, Oizumi K, Ohshima Y, Honda E. Cerebrospinal fluid ferritin in glioblastoma: Evidence for tumor synthesis. Journal of Neuro-Oncology. 1998;40(1):47-50.
[10] Schwartzbaum JA, Cornwell DG. Oxidant stress and glioblastoma multiforme risk: serum antioxidants, gamma-glutamyl transpeptidase, and ferritin. Nutr Cancer. 2000;38(1):40-9.[11] Lotz J, Hafner G, Prellwitz W. Reference Values for a Homogeneous Ferritin Assay and Traceability to the 3rd International Recombinant Standard for Ferritin (NIBSC Code 94/572). Clinical Chemistry and Laboratory Medicine1999. p. 821.

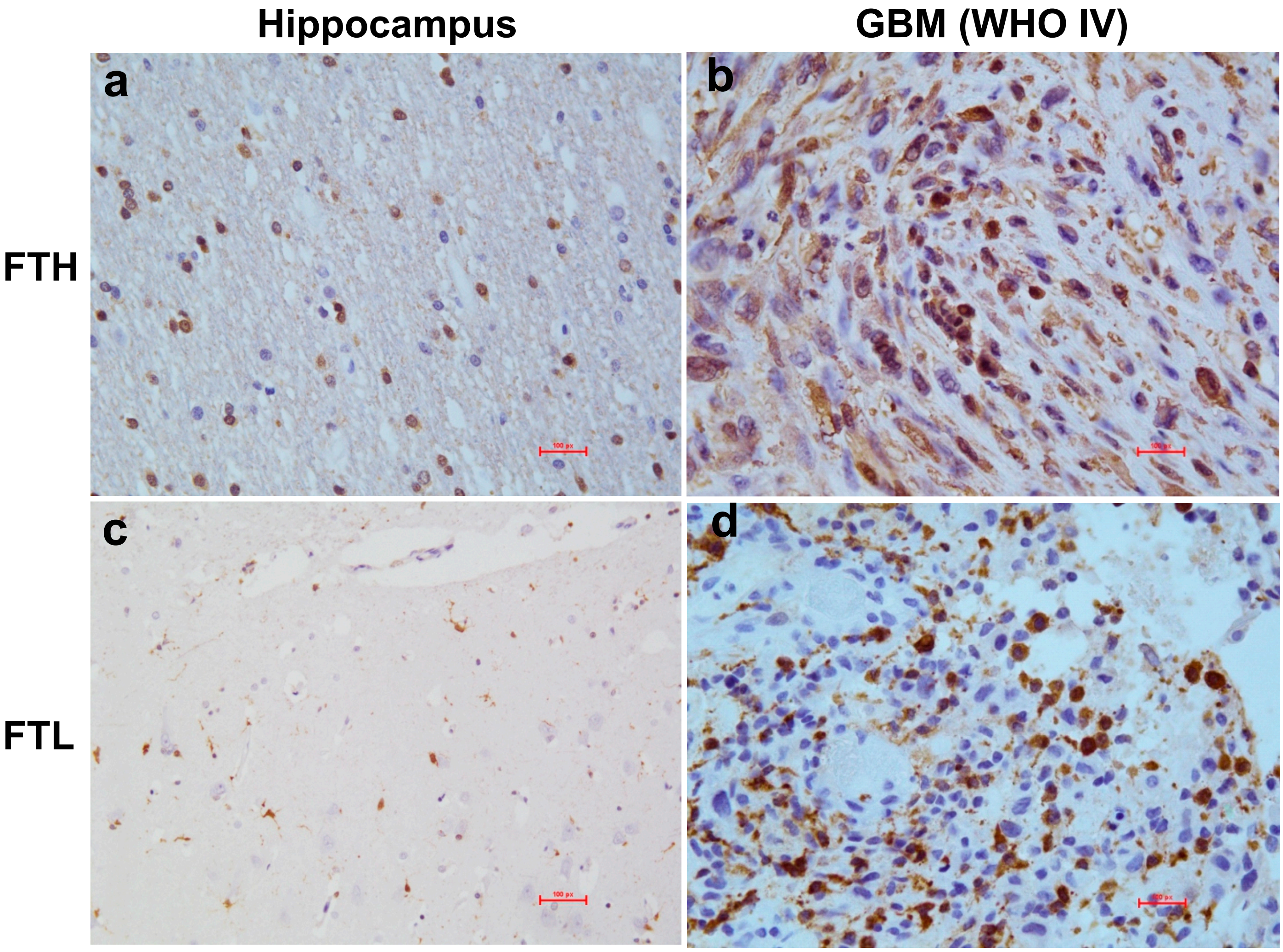

Supplemental Fig. S1. Immunohistochemical staining of hippocampus and GBM (WHO IV) tissue for ferrtin subunits FTH (a,b) and FTL (c,d). Formalin-fixed paraffin-embedded (FFPE) specimens were prepared from resected brain tumour tissue. 2-4 µm sections were cut and placed on adhesive microscope slides and subsequently dried for one hour at 60° C. De-paraffination, rehydration and antigen retrieval was carried out following standard operating procedures using diluted Target Retrieval Solutions pH6 and pH9 (EnVisionTM FLEX Target Retrieval Solution High and Low 50x, Dako, Vienna, Austria) pre-heated to 84°C. Slides were boiled for 17 min in the pH9 buffer for ferritin L-chain (FTL) staining and 20 min at 96°C in the pH6 buffer for ferritin H-chain (FTH). Immunohistochemical staining was performed using the Dako Autostainer Plus following standard operating procedures. The employed primary antibodies were anti-human FTL specific polyclonal goat antibody (LSBio LS-B4383, Eubio, Vienna, Austria) used at a dilution of 1:800 and anti-FTH specific rabbit anti human monoclonal antibody (LSBio LS-C105404, Eubio, Vienna, Austria) used at a dilution of 1:200. HRP-conjugated EnVisionTM FLEX secondary antibody (Dako) was used at a dilution of 1:25. EnVisionTM FLEX DAB +/- chromogen was diluted in EnVisionTM FLEX substrate buffer and the reaction stopped in EnVisionTM FLEX Peroxidase Blocking Reagent (Dako). For the anti-ferritin L chain, the anti-goat linker was used (EnVision+TM Dual Link System-HRP 1:400, Dako). All slides were counterstained with hematoxylin, differentiated, dehydrated and mounted in Tissue Tek Glass Mounting Medium following standard operating procedures. The labelling index (i.e., the percentage of immunoreactive cells) of the specimens was determined according to the uniform method for quantification of the hormone receptor content of Remmele and Stegner (Remmele W, Stegner HE. Pathologe. 1987;8:138-40) The percentage of immunopositive cells related to the total number of cells was evaluated in 4 randomly selected visual fields. Specimens of the human hippocampus served as reference Four independent areas were evaluated per sample for immunoreactive cells yielding an average of >1000 scored cells per tumour specimen.

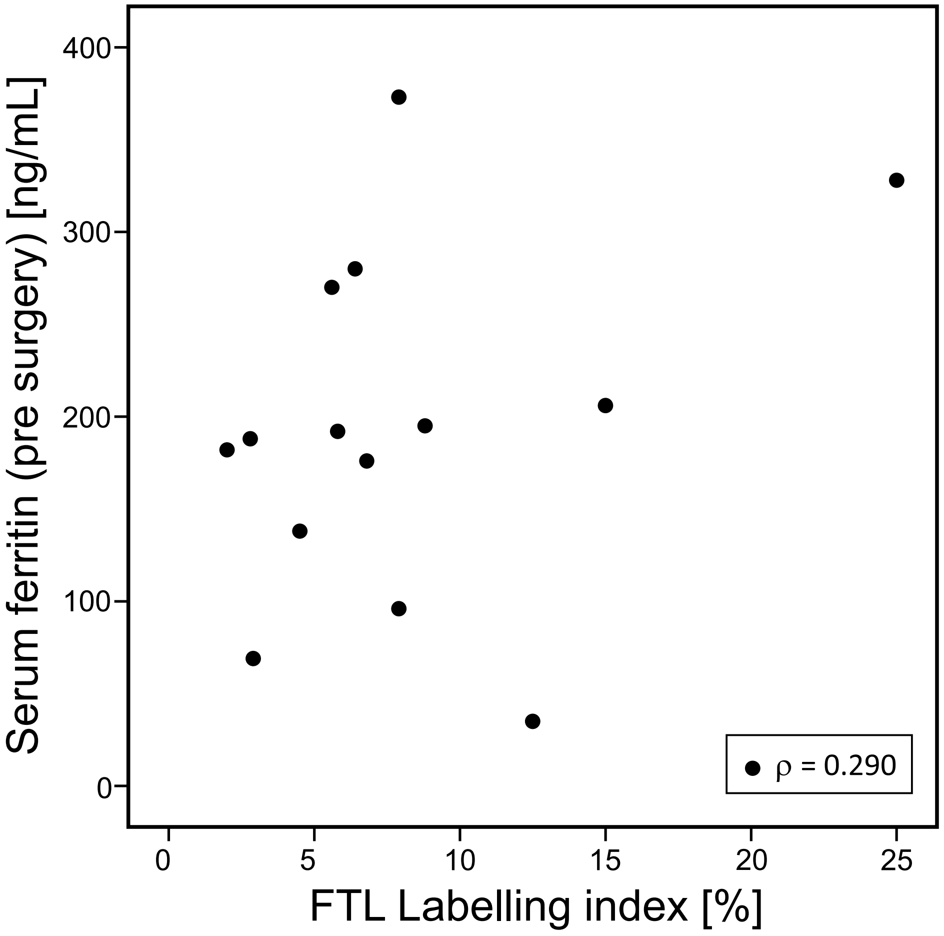

Supplemental Fig. S2. Correlation between the FTL labelling index of resected tumour tissue and the corresponding SF levels in GBM patients showing SF levels within the 95 percentile of the reference range (RefR cohort) of the Cobas Tina Quant FERR4 ® (ROCHE Diagnostics). Insert: Spearman's rho correlation coeefficient).
